# Latent *Toxoplasma gondii* infections are associated with elevated biomarkers of inflammation and vascular injury

**DOI:** 10.1186/s12879-021-05882-6

**Published:** 2021-02-18

**Authors:** Andrey I. Egorov, Reagan R. Converse, Shannon M. Griffin, Jennifer N. Styles, Elizabeth Sams, Edward Hudgens, Timothy J. Wade

**Affiliations:** 1 United States Environmental Protection Agency, Office of Research and Development, EPA, MD 58-C, 109. T.W. Alexander Drive, Research Triangle Park, NC 27711 USA; 2 United States Environmental Protection Agency, Office of Research and Development, Cincinnati, OH USA; 3 Gillings School of Global Public Health, Environmental Sciences and Engineering Department, University of North Carolina at Chapel Hill, Chapel Hill, NC USA

**Keywords:** *Toxoplasma gondii*, Seroprevalence, Biomarkers, Vascular injury

## Abstract

**Background:**

*Toxoplasma gondii* is a protozoan parasite that infects cats as definitive hosts and other warm-blooded animals including humans as intermediate hosts. It forms infectious cysts in the brain, muscle and other tissues establishing life-long latent infection. Approximately 10% of the US population is infected. While latent infections are largely asymptomatic, they are associated with neurological deficits and elevated risks of neuropsychiatric diseases.

**Methods:**

This cross-sectional epidemiological study investigated associations of *T. gondii* infections with biomarkers of inflammation and vascular injury: soluble intercellular adhesion molecule 1 (ICAM-1), soluble vascular cell adhesion molecule 1 (VCAM-1), C-reactive protein (CRP), and serum amyloid A (SAA). Serum samples from 694 adults in the Raleigh-Durham-Chapel Hill, North Carolina metropolitan area were tested for IgG antibody response to *T. gondii*, and for the above biomarkers using commercially available assays.

**Results:**

*T. gondii* seroprevalence rate in this sample was 9.7%. Seropositivity was significantly associated with 11% (95% confidence limits 4, 20%) greater median levels of VCAM-1 (*p* = 0.003), and marginally significantly with 9% (1, 17%), and 36% (1, 83%) greater median levels of ICAM-1, and CRP, respectively (*p* = 0.04 for each) after adjusting for sociodemographic and behavioral covariates, while the 23% (− 7, 64%) adjusted effect on SAA was not statistically significant (*p* = 0.15).

**Conclusions:**

Latent infections with *T. gondii* are associated with elevated biomarkers of chronic inflammation and vascular injury that are also known to be affected by ambient air pollution.

## Background

*Toxoplasma gondii* is a protozoan parasite that infects felines as definitive hosts and other warm-blooded animals as intermediate hosts, including humans as accidental or dead-end hosts [38]. Feces of infected cats contain *T. gondii* oocysts with infectious sporozoites that can remain viable in soil for years [16, 34]. Intermediate hosts including livestock can become infected through ingestion of oocyst contaminated soil. The parasite forms a life-long infection in the hosts persisting in bradyzoite-containing infectious tissue cysts in muscles, the central nervous system, and other tissues. The parasite completes its life cycle when cats ingest tissues of infected intermediate hosts. Humans become infected mainly through consumption of raw or undercooked meat or accidental ingestion of environmental oocysts [30].

Serum IgM response appears for a short period after initial infection. Serum IgG antibodies to this parasite usually peak in 2 to 3 months and then gradually decline to a lower but still detectable level characteristic of a chronic infection [46]. Therefore, serum IgM and IgG tests are typically used for detecting and differentiating acute and latent *T. gondii* infections [31, 32]. The latest available national US surveillance shows 13.2% IgG seroprevalence in individuals older than 5 years of age [31].

Clinical symptoms of new infections in humans include ocular disease, lymphadenitis, encephalitis, and myocarditis [24]. However, about three-quarters of new infections in healthy individuals are asymptomatic [58]. *T. gondii* infection during pregnancy is especially dangerous as the parasite can cause spontaneous abortion or severe neurological abnormalities in a newborn [30]. *T. gondii* infections in rodents and primates have been associated with behavioral modifications that make them more vulnerable to predation by felines [29, 42, 57]. Behavioral abnormalities in infected animals have been associated with chronic neuroinflammation [10, 27].

Latent *T. gondii* infections in humans have been associated with various adverse neuropsychiatric outcomes including suicide and increased risk of traffic accidents [50], schizophrenia and bipolar disorder [11, 13], obsessive compulsive disorder [39], and increased aggression and impulsivity [14]. Other studies linked latent infections with an increased risk of type 2 diabetes [36], rheumatoid arthritis [28] and Alzheimer’s disease [40].

Latent *T. gondii* infections have also been linked with immune activation and subtle neurophysiological changes [53, 55]. Previous research demonstrated associations between *T. gondii* infection and elevated serum levels of markers of inflammation, dyslipidemia, and cardiovascular events, specifically endothelial adhesion molecules, ICAM-1, VCAM-1, as well as pro-inflammatory cytokines [21, 23, 55].

Our previous epidemiological study in 206 adults in North Carolina demonstrated that *T. gondii* seropositivity was associated with an elevated allostatic load – a composite measure of physiological dysregulation comprised of 15 biomarkers of neuroendocrine, metabolic, immune and endothelial function including ICAM-1, VCAM-1, CRP and SAA [21]. While associations with many individual biomarkers were positive, only a few of these effects including increased levels of myeloperoxidase (the enzyme involved in immune response to the parasite), proinflammatory cytokine IL-6, and VCAM-1 were statistically significant.

To our knowledge, this was the first epidemiolocal study demonstrating an association between latent *T. gondii* infection and elevated serum level of VCAM-1. However, as that study explored associations with many biomarkers, a chance finding due to multiple testing could not be ruled out. The study population included only 17 seropositive and 189 seronegative individuals. While the relatively small sample size was sufficient for analysis of allostatic load - a statistically powerful approach simultaneously utilizing data on multiple biomarkers to assess systemic effects - a bigger study was necessary to further investigate associations with individual biomarkers.

The objective of the present study was to test associations of latent *T. gondii* infections with individual biomarkers of inflammation and vascular injury in a larger sample of adult individuals. The study involved analysis of four biomarkers that have been linked to *T. gondii* in previous in vivo or in vitro studies and that are known predictors of adverse health outcomes in humans: ICAM-1, VCAM-1, CRP and SAA. Adhesion molecules ICAM-1 and VCAM-1 are released into circulation in response to inflammation by vascular endothelial cells. They mediate leukocyte adherence to the vascular endothelium and transmigration. Previous research suggested that *T. gondii* exploits these natural cell trafficking pathways to cross cellular barriers including the blood-brain barrier [3, 25]. CRP and SAA are biomarkers of inflammation. Elevated levels of ICAM-1, VCAM-1, CRP and SAA have been associated with coronary artery disease, cancer and psychiatric disorders including schizophrenia [4, 9, 33, 41, 55, 60].

## Methods

### Study population

Serum samples and questionnaire data were collected as part of a cross-sectional population-based observational study of chronic infections in the Raleigh-Durham-Chapel Hill metropolitan area in North Carolina in 2013. The study protocol was approved by the Institutional Review Board of the University of North Carolina at Chapel Hill (UNC IRB ref. # 12-2600).

The study population was comprised of two subsets of adults (individuals at least 18 years of age). The first subset was a convenience sample of the adult population of Raleigh-Durham-Chapel Hill, NC metropolitan area (n_1_ = 352) recruited by the United States Environmental Protection Agency (EPA). The study was advertised on-line and by displaying study posters at local venues. All participants signed informed consent forms prior to data collection. Blood samples and questionnaire data were collected at the EPA Human Studies Facility in Chapel Hill, NC. Approximately 200 samples from this subset have been analyzed for biomarkers of inflammation and endothelial function/vascular injury, and for anti-*T. gondii* IgG responses under previous research projects on urban green spaces and allostatic load [22], and green spaces, *T. gondii* infections and allostatic load [21].

The second subset of adult individuals (n_2_ = 351) was recruited for the Sample Collection Registry for Quality Control of Biological and Environmental Specimens and Assay Development and Testing study protocol number 10-E-0063, ClinicalTrials.gov identifier NCT01087307 at the National Institute of Environmental Health Sciences (NIEHS). Recruitment and data collection were conducted at the Research Triangle Park, NC facility of NIEHS. Aliquots of serum samples and questionnaire data from this study were shared with EPA. Home addresses or geographic coordinates of study participants were not accessible to EPA researchers.

The present study involved the use of the same EPA study data that were analyzed previously [21] as well as questionnaire and biomarker data on approximately 500 additional individuals. The total sample size of approximately 700 individuals was expected to be sufficient for analysis of associations between latent *T. gondii* infections and individual biomarkers of vascular injury. This combined dataset has already been used in the analysis of IgG responses to cytomegalovirus (CMV), and associations between anti-CMV antibody responses and biomarkers of health [49].

### Serological tests

Serum samples from study participants were tested for anti-*T. gondii* IgG responses using VIR-ELISA Anti-Toxo IgG assay kits (VIRO-IMMUN Labor-Diagnostika GmbH, Oberursel, Germany). The assay included serial calibrators with known concentrations in International Units (IU), and controls. The results were dichotomized as seropositive or seronegative using the 50 IU calibrator as a cut-off as described previously [21]. Serum levels of SAA, CRP, VCAM-1, and ICAM-1 were analyzed using a commercially available electrochemiluminescence Meso Scale Discovery (MSD) quadruplex Vascular Injury Panel 2 microplate assay (cat# K15198D, MSD, Rockville, Maryland) following manufacturer’s instructions. Appropriate controls and serially diluted standards with known concentrations of each analyte were assayed on each plate. Responses were measured using an MSD QuickPlex SQ 120 instrument. Concentrations of the analytes were estimated from four-parameter logistic regression models fitted to data on serially diluted standards. Serum samples were also tested for IgG antibodies to CMV using a commercially available ELISA assay as described previously [49].

### Questionnaire and anthropometrical data

Different questionnaires were used to collect sociodemographic and behavior data on the two subsets of participants recruited at EPA and NIEHS. While basic sociodemographic data, such as age, gender, race, and ethnicity were collected consistently, some questions on relevant social and behavioral factors were formulated differently. The current analysis utilized only those questionnaire data that were collected consistently and could be combined into a pooled dataset. Data on education, occupation and income, as well as residential addresses, were not available for the subset of individuals recruited at NIEHS.

Body mass index (BMI) values were estimated using weight and height measurements performed in both subsets by health professionals.

### Statistical data analysis

Statistical analysis was conducted using SAS version 9.4 software (SAS Institute, Cary, NC). Univariate analysis of associations between demographic, behavioral and environmental factors and *T. gondii* infections was conducted using the Chi-square Wald test for binary and nominal variables and the Cochran-Armitage test for trend for ordinal variables. Missing values were excluded from the univariate analysis.

Analysis of missingness types and patterns [18] was conducted using logistic regression and chi-squared tests. For multivariate regression analysis, missing data on sociodemographic and behavioral covariates were imputed using the SAS procedure *mi* utilizing the multivariate discriminant function method with the FCS (Fully Conditional Specifications) statement specifying a multivariate imputation by chained equations method for nominal Missing at Random (MAR) data with arbitrary missingness pattern [18, 59].

Regression models were fitted to the full dataset with imputed missing data using the SAS procedure *genmod*. A multivariate predictive regression model of *T. gondii* seropositivity was developed using all available sociodemographic and behavioral covariates applying the backward stepwise selection method.

Log-transformed biomarker data were used in pair-wise correlation analysis and in regression analyses. Three sets of regression models were developed to investigate the effects of *T. gondii* seropositivity on biomarkers: (1) base models adjusting for age; (2) multivariate models including additional sociodemographic and behavioral covariates which were selected for each biomarker separately using the bi-directional elimination approach; and (3) full models including all covariates from the second model plus CMV serostatus and self-reported diabetes.

## Results

### Descriptive statistics and univariate analysis

The final dataset involved data on 694 of 703 (99%) study participants. Among them, there were 206 (30%) individuals whose data were used in our previous publication on *T. gondii* infections and allostatic load [21]. Leftover serum samples from the remaining 9 individuals could not be analyzed for all biomarkers due to various technical reasons, such as limited sample volumes.

The mean age of participants in the entire sample was 41.0 years (standard deviation 14.3 years and range from 18 to 85 years) with means of 38.2 and 43.8 years in the EPA and NIEHS subsets, respectively. The racial and ethnic compositions of the EPA and NIEHS subsets were similar with 63.4 and 63.9% of non-Hispanic White individuals, respectively. The overall *T. gondii* seroprevalence in this study population was 9.7% (Table 1). In the univariate analysis, the seroprevalence rate was significantly higher in the subset of participants recruited at NIEHS (12.1%) than in the subset recruited at EPA (7.2%). Seropositivity was strongly associated with age (*p* <  0.0001, Cochran-Armitage test for trend) increasing from 2.4% in the 18 to 29 years of age group to 22.1% in the 60 to 85 years of age group. BMI, gender, race, Hispanic ethnicity, type of drinking water supply and diabetes status were not associated with *T. gondii* seropositivity in univariate Chi-square tests. History of living on a farm and current smoking were both strongly associated with *T. gondii* seropositivity. As most study participants were either white (54%) or black (27%), race data were dichotomized as white vs. all other races for imputation of missing values.

**Table 1:** Descriptive statistics of the study population and univariate analysis of predictors of *T. gondii* seropositivity

Variable	Level	Participants, N (% of total)	***T. gondii*** seropositive, N (row %)	***p*** value
All participants		694 (100%)	67 (9.7%)	na
Subset of participants by recruitment site	EPA	348 (50%)	25 (7.2%)	
	NIEHS	346 (50%)	42 (12.1%)	0.03
Age (years)	18-29	206 (30%)	5 (2.4%)	
	30-39	138 (20%)	8 (5.8%)	
	40-49	130 (19%)	18 (14%)	
	50-59	152 (22%)	21 (14%)	
	60-85	68 (10%)	15 (22%)	< 0.0001*
BMI (kg/m2)	Underweight (< 18.5)	7 (1.0%)	1 (14%)	
	Normal Weight (18.5 - 24.9)	226 (33%)	18 (8.0%)	
	Overweight (25.0 - 29.9)	209 (30%)	21 (10%)	
	Obese (≥ 30)	252 (36%)	27 (11%)	0.7
Gender	Male	264 (38%)	28 (11%)	
	Female	430 (62%)	39 (9.1%)	0.5
Race	White	376 (54%)	30 (8.0%)	
	Black or African American	187 (27%)	22 (12%)	
	American Indian or Alaska Native	2 (0.3%)	0 (0%)	
	Asian or Pacific Islander	9 (1.3%)	0 (0%)	
	Other	17 (2.4%)	0 (0%)	0.3
	Not reported	103 (15%)	15 (15%)	
Ethnicity	Non-Hispanic	558 (80%)	50 (9.0%)	
	Hispanic	34 (4.9%)	3 (8.8%)	1
	Not reported	102 (15%)	14 (14%)	
History of living on a farm	No	539 (78%)	43 (8.0%)	
	Yes	151 (22%)	24 (16%)	0.004
	Not reported	4 (1.9%)	0 (0%)	
Drinking water supply	Private well	115 (17%)	8 (7.0%)	
	Public system	573 (83%)	59 (10%)	0.3
	Not reported	6 (0.9%)	0 (0%)	
Self-reported cardiovascular disease	No	578 (83%)	55 (9.5%)	
	Yes	8 (1.2%)	0 (0%)	0.4
	Not reported	108 (16%)	12 (11%)	
Self-reported diabetes	No	634 (91%)	59 (9.3%)	
	Yes	57 (8.2%)	7 (12%)	0.5
	Not reported	3 (0.4%)	1 (33%)	
Smoking habits	Non-smoker	531 (77%)	42 (7.9%)	
	Current smoker	159 (23%)	25 (16%)	0.003
	Not reported	4 (0.6%)	0 (0%)	

^*^ Cochran-Armitage test for trend was used

Descriptive statistics on concentrations of biomarkers are presented in Table 2. There were no biomarker values below limits of quantitation in this dataset. Biomarker data were approximately log-normally distributed. Pearson correlation analysis among log-transformed biomarkers, age and BMI (Table 3) shows the strongest correlations between CRP and SAA (*r* = 0.61), and between VCAM-1 and ICAM-1 (*r* = 0.50). Age was positively correlated with all biomarkers with correlation coefficients ranging from 0.17 to 0.21 (*p* <  0.0001 for all) and BMI. In turn, BMI had strong, highly significant correlations with CRP (*r* = 0.53), SAA (*r* = 0.37), and ICAM-1 (*r* = 0.28), while its correlation with VCAM-1 was very weak (*r* = 0.06) and not significant.

**Table 2:** Concentrations of inflammation and vascular injury biomarkers, ng/mL

Biomarker	Min	10th pctl	Median	Mean	SD	90th pctl	Max
VCAM-1	272	465	658	692	242	946	4136
ICAM-1	186	348	500	545	213	788	2696
CRP	45	285	2268	5575	11,568	13,426	143,374
SAA	44	448	1723	5708	26,340	93,278	441,436

**Table 3:** Pearson correlations among log-transformed biomarkers, age and BMI data

	log (VCAM-1)	log (ICAM-1)	log (CRP)	log (SAA)	Age
**log (VCAM-1)**	1				
**log (ICAM-1)**	0.50	1			
**log (CRP)**	0.17	0.44	1		
**log (SAA)**	0.21	0.35	0.61	1	
**Age**	0.15	0.21	0.21	0.20	1
**BMI**	0.06	0.28	0.53	0.37	0.22

### Imputation of missing values of explanatory variables

All explanatory variables except age, BMI and gender contained some missing data which are denoted as “Not reported” in Table 1. Data on race and ethnicity were missing most often (103 and 102 individuals, respectively). There were 95 individuals who failed to report both race and ethnicity. For regression analysis, race and ethnicity data were merged in a single binary variable (White non-Hispanic individuals vs. all others) as described in the Methods section. Other explanatory variables had an arbitrary missingness pattern with missing data occurring in any variable for any participant in a random fashion. Analysis of missingness type demonstrated that assumptions for Missing Completely at Random (MCAR) were not satisfied as the likelihood of missingness in some variables was not independent from the known values of other variables. For example, race and ethnicity data were significantly more likely to be missing in CMV seropositive individuals (not shown). As missing values were unknown, it was not possible to test whether the missingness type is Missing at Random (MAR, assuming that the likelihood of missingness independent from missing values) or Missing Not at Random (MNAR) when this assumption is violated. As it has been shown that violations of the MAR assumption do not seriously distort parameter estimates and formulating multivariable imputation models usually makes the missingness type ignorable [18], missing data imputations were conducted using SAS procedure *mi* with the FCS statement for MAR data as described in the Methods section.

### Predictors of *T. gondii* seropositivity and its association with smoking status

In multivariate regression analysis, *T. gondii* seropositivity was significantly positively associated with age, smoking, and history of living on a farm, and negatively associated with having a private water well, a proxy for less densely populated suburban and rural locations (Table 4). Participants recruited at NIEHS were, on average, older (43.8 vs. 38.2 years), had a higher prevalence of smoking (24.4% vs. 21.7%), more frequently reported living on a farm in the past (23.0% vs. 20.8%), and less frequently reported having a private water well (13.9% vs. 19.5%) compared to participants recruited at EPA. After adjusting for these covariates, the effect of recruitment site on *T. gondii* seropositivity was no longer significant (not shown). Self-reported consumption of undercooked beef or pork within the previous 3 months was also not a significant predictor of *T. gondii* seropositivity in this analysis (not shown). Data on consumption of lamb and game meat were not collected. CMV seropositivity was not significantly associated with *T. gondii* seropositivity after adjusting for age and other covariates.

**Table 4:** Predictors of *T. gondii* seropositivity

Predictor	Level	Adjusted OR (95% CL)
Age (per 10-year increase)	NA	1.81 (1.48, 2.21)
Current smoker	No	Reference
	Yes	2.62 (1.49, 4.63)
Ever lived on a farm	No	Reference
	Yes	1.92 (1.08, 3.41)
Tap water source	Public supplies	Reference
	Private well	0.44 (0.20, 0.99)

Smoking could be a proxy for unmeasured behavioral and sociodemographic predictors of *T. gondii* infections. Alternatively, *T. gondii* might make infected individuals more likely to become smokers (reverse causation). Additional analysis using smoking status as an outcome variable and *T. gondii* serostatus as a predictor demonstrated that odds ratio of smoking in seropositive individuals compared to seronegative controls was 2.2 (1.2, 3.9) after adjusting for age, race, gender and history of living on a farm.

### Latent *T. gondii* infection as a predictor of vascular injury biomarkers

In these analyses, *T. gondii* serostatus was modeled as a dichotomous predictor variable while outcome variables were individual biomarkers. The results in Table 5 show that levels of three biomarkers, VCAM-1, ICAM-1 and CRP were positively associated with *T. gondii* seropositivity. The most significant adjusted effect estimates were observed for VCAM-1. The strongest effect, 12% (4, 21%) increase in the median level of VCAM-1 was observed in Model 1 after adjusting for age only. Adjusting for sociodemographic covariates in Model 2, and also for CMV serostatus and self-reported diabetes in Model 3 had very minor impacts on the effect estimates reducing them to 11% (4, 20%) and 11% (4, 19%), respectively. The associations of seropositivity with ICAM-1 and CRP were the strongest in Model 1 at 15% (6, 26%) and 53% (7, 118%), respectively. In Model 2, adding sociodemographic covariates reduced them substantially to 9% (1, 17%) for VCAM-1 and 36% (1, 83%) for CRP. In Model 3, the effect estimates for these two biomarkers became only marginally significant (*p* = 0.05 for each) after adjusting for CMV serostatus and diabetes. In all three models, the effect estimates for SAA were positive but not statistically significant.

**Table 5.** Associations between *T. gondii* seropositivity and vascular injury biomarkers, percent change in median biomarker level adjusted for covariates. Sociodemographic covariates in regression models for VCAM-1 and CRP were: gender, race, smoking, BMI, water supply type; for ICAM-1: race, smoking, BMI, water supply type; and for SAA: gender, race, and history of living on a farm

Model	*T. gondii* serostatus	VCAM-1		ICAM-1		CRP		SAA	
		Effect estimate (95% CI)	***p*** value	Effect estimate (95% CI)	***p*** value	Effect estimate (95% CI)	***p*** value	Effect estimate (95% CI)	***p*** value
Model 1. Adjusted for age	Negative	Reference		Reference		Reference		Reference	
	Positive	12 (4, 21)	0.002	15 (6, 26)	0.001	53 (7, 118)	0.02	21 (−11, 64)	0.22
Model 2. Adjusted for age and sociodemographic covariates	Negative	Reference		Reference		Reference		Reference	
	Positive	11 (4, 20)	0.003	9 (1, 17)	0.04	36 (1, 83)	0.04	23 (−7, 64)	0.15
Model 3. Adjusted for model 2 covariates, CMV serostatus and diabetes.	Negative	Reference		Reference		Reference		Reference	
	Positive	11 (4, 19)	0.004	8 (0, 17)	0.05	34 (0, 81)	0.05	22 (−8, 62)	0.18

## Discussion

This cross-sectional epidemiological study demonstrated that *T. gondii* IgG seropositivity (a marker of latent infection) was associated with elevated serum levels of three vascular injury and inflammation biomarkers: VCAM-1, ICAM-1 and CRP (the latter two associations were only marginally significant). The study involved 694 adult individuals, 67 of whom were seropositive to *T. gondii*. Compared to our previous study in the same area [21], this new study had almost three-and-a-half times bigger sample size and four times as many seropositive individuals.

The observed 9.7% seroprevalence of *T. gondii* in this study of adults was comparable with the 13.2% national estimate of unadjusted seroprevalence in the 2009-2010 NHANES study which involved individuals of at least 5 years of age [31]. As previous studies showed a declining *T. gondii* seroprevalence in the US, it is likely that the current national seroprevalence rate is below the 2009-2010 estimate.

This study involved two subsets of participants recruited at EPA and NIEHS facilities located in the Raleigh-Durham-Chapel Hill, NC metropolitan area approximately 17 km apart. Participants were recruited using convenience sampling approaches. As a result, the study population was different from the source population due to the greater willingness of certain categories of people, such as women, to participate. The EPA recruitment site was located on the campus of University of North Carolina in Chapel Hill, a city of about 60,000 residents with a high proportion of university graduates. The NIEHS recruitment site was located closer to the city of Raleigh (the capital of North Carolina, population 250,000); it is likely that the NIEHS subset included more residents of Raleigh, but the residence data were not available for this analysis. The observed difference between *T. gondii* seropositivity rates in NIEHS and EPA subsets was largely explained by their sociodemographic compositions and life histories. Although the pooled study population was more representative of the diverse sociodemographic conditions in central North Carolina than each subset, the seroprevalence estimate in this study might not accurately represent *T. gondii* seroprevalence in the general population of this area.

In the present study, history of living on a farm was a predictor of *T. gondii* seropositivity. This finding is consistent with previously published results [44]. Other common risk factors for *T. gondii* infections such as eating undercooked meat, contacts with soil and owning a cat were detected in our previous analysis [21], but could not be confirmed in the present study given discrepancies in how questions in EPA and NIEHS questionnaires regarding cat ownership, eating raw or undercooked meat and handling soil were formulated. It should also be noted that data on consumption of lamb and game meat, which may be important sources of *T. gondii* infections, were not collected.

In this study, smoking and *T. gondii* seropositivity were positively associated. Previous analysis of National Health and Nutrition Examination Survey (NHANES) data in the US demonstrated that latent *T. gondii* infections were associated with a slightly reduced likelihood of self-reported tobacco usage as well as significantly reduced usage of heroin and methamphetamine [5]. The effects on tobacco usage, however, were inconsistent between different rounds of NHANES survey and across income and education categories with *T. gondii* being a risk factor for tobacco usage in subgroups with high income and high education. Further research is needed to elucidate potential relationships between *T. gondii* and smoking.

The effects of latent *T. gondii* infections on vascular injury and inflammation biomarkers observed in this study reflect underlying pathophysiological processes, which may be triggered by a periodic rupture of the tissue cysts and release of bradyzoites that happens spontaneously in intermediate hosts [20]. In immunocompetent individuals, this triggers immune system activation that clears the released bradyzoites. Adhesion molecules VCAM-1 and ICAM-1 are released into circulation by vascular endothelial cells in response to inflammation. They mediate leukocyte adherence to the vascular endothelium and transmigration. In vitro experiments also demonstrated that expressions of ICAM-1 and VCAM-1 are upregulated in *T. gondii* infected bovine endothelial cells [52]. The parasite has been shown to use ICAM-1 to cross the blood-brain barrier and other endothelial barriers during the acute phase of infection [3, 25]. The role of ICAM-1 during the latent infection stage remains to be characterized. VCAM-1 is essential for controlling *T. gondii* infection [17, 47] and infections are associated with elevated serum levels of VCAM-1 in rodents [55]. Chronically elevated levels of these adhesion molecules are linked with the development of atherosclerosis [9, 35, 60]. Previous research has also associated elevated levels of VCAM-1 and ICAM-1 with schizophrenia [41]. CRP is a marker of acute inflammation which is elevated in *T. gondii*-infected animals [55]. Chronically elevated CRP is also associated with cardiovascular disease [33, 60], major depressive disorder [26], generalized anxiety disorder [15], and schizophrenia [37], as well as greater severity of symptoms in patients with schizophrenia [12].

Previous epidemiological studies have demonstrated associations between *T. gondii* seropositivity and ICAM-1 [23], VCAM-1 [21] and CRP [8, 51], consistent with our new findings. The positive association between latent *T. gondii* infection and serum levels of VCAM-1 in humans was demonstrated for the first time in our previous publication [21] and confirmed in the present analysis.

Our analysis of data on the same set of individuals [49] also demonstrated that CMV IgG seropositivity or the intensity of anti-CMV IgG responses were associated with elevated levels of the same vascular injury biomarkers, VCAM-1, ICAM-1 and CRP. The present study showed that the effects of *T. gondii* on these biomarkers were largely independent from the effects of CMV. Adjusting for CMV serostatus did not have substantial impacts on effects estimates for *T. gondii* seropositivity (Table 5, models 2 and 3): effect estimates for VCAM-1, ICAM-1, and CRP were reduced from 11.4 to 11.2% (2% reduction of the effect size), from 8.6 to 8.2% (5% reduction), and from 35.8 to 34.2% (4% reduction), respectively. It should be noted, however, that the latter adjusted effects were only marginally significant.

Both CMV and *T. gondii* infections have been linked with psychiatric disorders suggesting potential overlapping biological pathways to detrimental health effects [11]. Chronic inflammation plays an important role in pathophysiology of psychiatric disorders but specific cause-effect mechanisms underlying this association remain to be elucidated [45]. Previous research demonstrated that *T. gondii* infection causes neural damage and reactive tissue repair in mice similar to those observed in the brain of schizophrenia patients, and that both *T. gondii* infections in mice and schizophrenia are associated with elevated levels of CRP and VCAM-1 [55]. While there is accumulating evidence of immune response activation sustaining inflammation in *T. gondii*-infected individuals as a pathway to behavioral changes, neurological deficit and psychiatric disorders, specific molecular mechanisms of these effects remain to be characterized [56].

Furthermore, previous studies also demonstrated that exposure to common air pollutants is associated with increased serum levels of CRP, ICAM-1 and VCAM-1 in humans [6, 7], suggesting that *T. gondii*-infected individuals may be more susceptible to the effects of air pollution. Schizophrenia patients have elevated levels of these biomarkers [41], and exposure to common ambient air pollutants is associated with increased hospital admissions for schizophrenia and other psychiatric disorders [2, 19, 43] as well as atherosclerosis, thrombosis, and increased mortality in chronically exposed individuals [1, 54]. Further investigations of the interaction effects of *T. gondii* infections and exposure to environmental hazards on the risk of neuropsychiatric and systemic diseases are warranted.

This cross-sectional observational study could only demonstrate statistical associations; it was not designed to establish a cause-effect relationship. Some of the observed associations, especially those that were marginally significant, could be due to random effects. The observed associations need to be further validated in different populations and countries, particularly in South America and other areas where more virulent strains are circulating [48]. Another important limitation of this study is that only a limited set of sociodemographic and socioeconomic variables was available for statistical analysis in the combined dataset due to inconsistently formulated questions in EPA and NIEHS survey forms. Most importantly, individual and household level education and income data were not available in this study. As residential addresses were not available for the NIEHS subset, we could not use area level socioeconomic indicators as a proxy for individual socioeconomic status. However, our previous analysis did not find an association between education level and *T. gondii* seropositivity [21] suggesting that socioeconomic status was unlikely to confound the observed associations between seropositivity and biomarkers of inflammation and vascular injury in this study population. Due to the small sample size, this analysis was limited to contrasting biomarker levels in seropositive and seronegative individuals. A bigger study with a greater number of seropositive individuals would be needed to explore potential associations between the intensity of anti-*T. gondii* IgG responses in infected individuals and levels of biomarkers of inflammation and vascular injury.

## Conclusions

In this study *T. gondii* IgG seropositivity was significantly associated with an increased level of soluble VCAM-1 in serum and marginally significantly with two more serum biomarkers of vascular injury and inflammation, ICAM-1 and CRP. Exposure to air pollution is known to affect the same biomarkers leading to various adverse health outcomes. Further research is needed to assess potential interaction effects of latent *T. gondii* infections and exposure to environmental hazards on morbidity and mortality.

## Data Availability

The datasets used and/or analyzed during the current study are available from the corresponding author on reasonable request.

## Abbreviations

aOR: Adjusted Odds Ratio
CMV: Cytomegalovirus
CL: Confidence Limits
CRP: C-Reactive Protein
EPA: United States Environmental Protection Agency
ICAM-1: Intercellular Adhesion Molecule 1
IgG: Immunoglobulin G
IgM: Immunoglobulin M
MAR: Missing at Random
MCAR: Missing Completely at Random
MNAR: Missing Not at Random
NIEHS: National Institute of Environmental Health Sciences
SAA: Serum Amyloid A
VCAM-1: Vascular Cell Adhesion Molecule 1
